# ORAL CANCER SCREENING IN NON-INSTITUTIONALIZED OLDER ADULTS LIVING IN RURAL COMMUNITIES OF SOUTHEAST GEORGIA

**DOI:** 10.1093/geroni/igz038.3235

**Published:** 2019-11-08

**Authors:** Swaha Pattanaik, Bettye Apenteng, Adrienne L Cohen, Georgia Dounis, Raymona Lawrence

**Affiliations:** 1 Jiann Ping Hsu College of Public Health, Georgia Southern University, Statesboro, Georgia, United States; 2 Georgia Southern University, Statesboro, Georgia, United States; 3 University of Nevada, Las Vegas, Las Vegas, Nevada, United States

## Abstract

The older population in the United States is growing at an unprecedented rate. Oral diseases such as oral cancer can affect physical, psychological, and social well-being in older adults. Oral cancer screening can prevent development of the disease in high-risk individuals. The purpose of this research was to assess determinants of preventive oral health behavior including oral cancer screening in noninstitutionalized older adults living in rural/medically underserved communities of southeast Georgia. A mixed methods sequential explanatory design was used. Surveys were administered to 206 individuals aged 50 and older. Phone interviews were conducted with 22 individuals from the survey sample and 11 key informants. The majority of the participants (83.01%) said they had never been examined for oral cancer by a doctor or a dentist. Those who correctly recognized the most common sign of oral cancer were about three times more likely (OR=3.75; 95% CI: 1.04 – 13.50) to have had an exam for oral cancer (p=0.04). The survey participants who lived alone were more likely (OR = 4.39; 95% CI = 0.95 – 20.26) to have been examined for oral cancer (p = 0.05). During the interview, older adult participants rarely mentioned oral cancer with regards to an unhealthy mouth. The interview participants revealed that living alone gave them more time to pay attention to their health. For the older adults, prevention of oral diseases was grounded in the autonomy in their own behaviors, while the key informants saw more macro community and systems- level factors as the solution.

